# Failure of crizotinib based systemic treatment in ALK positive histiocytosis involving the central nervous system: a case report and literature review

**DOI:** 10.1186/s12887-022-03368-1

**Published:** 2022-05-25

**Authors:** Qiang He, Wenjie Zhang, Qiang Li

**Affiliations:** 1 Department of Neurosurgery, West China Hospital of Sichuan University, No. 37 Guoxue Road, Chengdu, 610041 Sichuan Province People’s Republic of China; 2 Department of Nuclear Medicine, West China Hospital, Sichuan University, No. 37, Guoxue Alley, Chengdu, 610041 Sichuan People’s Republic of China

**Keywords:** ALK-positive histiocytosis, Chemotherapy, Ttreatment, Suprasellar region

## Abstract

**Background:**

Among the histiocytic disorders, anaplastic lymphoma kinase (ALK)-positive histiocytosis emerged in 2008. As more and more cases of the novel entity are reported, our understanding of it is deepened. However, only a few cases with central nervous system (CNS) involvement have been reported. Furthermore, the lesion in the suprasellar region has not been documented.

**Case presentation:**

We presented a case of ALK-positive histiocytosis involving the suprasellar region of a one-year-and-four-month-old boy. Through clinical, neuropathological, and genomic analyses, the patient was diagnosed with ALK-positive histiocytosis. After lesions were resected he started treatment with a combination of the three compounds vincristine, prednisolone, and crizotinib, but they did not work. Cytarabine was then added as an additional chemotherapy drug for him, and the lesions in the brain and lungs were shrunk by combining treatment of crizotinib, dexamethasone, vincristine, and cytarabine according to the RECIST (esponse Evaluation Criteria In Solid Tumours).

**Conclusions:**

Additional adjuvant chemotherapy drugs are needed when ALK-inhibitor treatment is ineffective.

## Background

In 2008, JK Chan et al. [1] first described the anaplastic lymphoma kinase (ALK)-positive histiocytosis, which is a novel proliferation of morphologically distinctive histiocytes with a chromosomal translocation involving ALK. Since then, they have reported 10 cases of ALK-positive histiocytosis, including one affecting the cavernous sinus [2]. ALK-positive histiocytosis in the central nervous system (CNS) has been rarely reported. It is either part of multiple disseminated lesions or the only manifestation of localized disease [2–13]. In particular, the lesion in the suprasellar region has never been described.

In this study, we reported the case of a one-year-and-four-month-old boy who was diagnosed with ALK-positive histiocytosis involving the suprasellar region following clinicopathological, molecular, and next-generation sequencing (NGS) examinations. Further, the literature review was performed to identify the clinical characteristics of the entity involving CNS.

## Case presentation

**Fig. 1:**
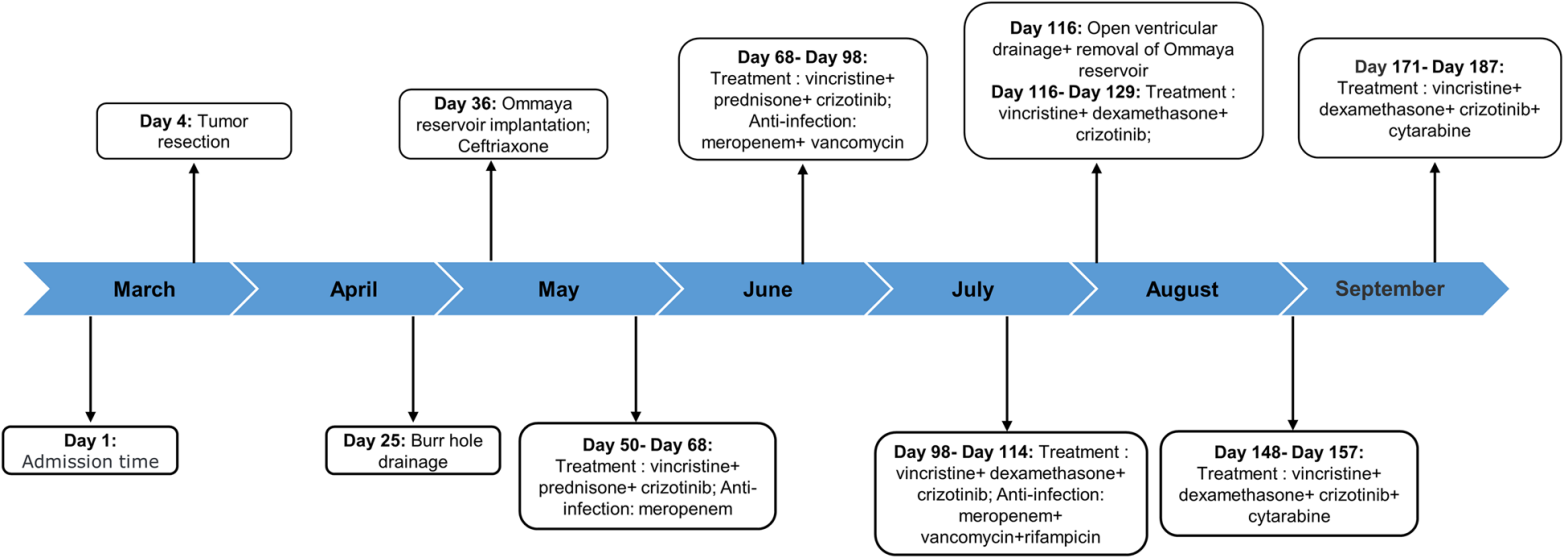
The timeline of patient treatment

The timeline of the treatment is shown in Fig. 1.

The patient was a one-year-and-four-month-old boy who was admitted for rapid weight loss and difficulty in walking for three months. A magnetic resonance imaging (MRI) of the head revealed a lesion in the suprasellar region. The result of the neurological examination showed that the fontanelle was slightly prominent. The head circumference was within normal limits. The patient could walk slowly with the help of his parents but not walk independently.

During pregnancy, the imaging examination of the mother of the patient showed no abnormalities. The patient and his family members did not have a similar illness or any other abnormal medical history. No abnormal phenomena were observed at birth. Indicators in routine child health care were normal.

**Fig. 2:**
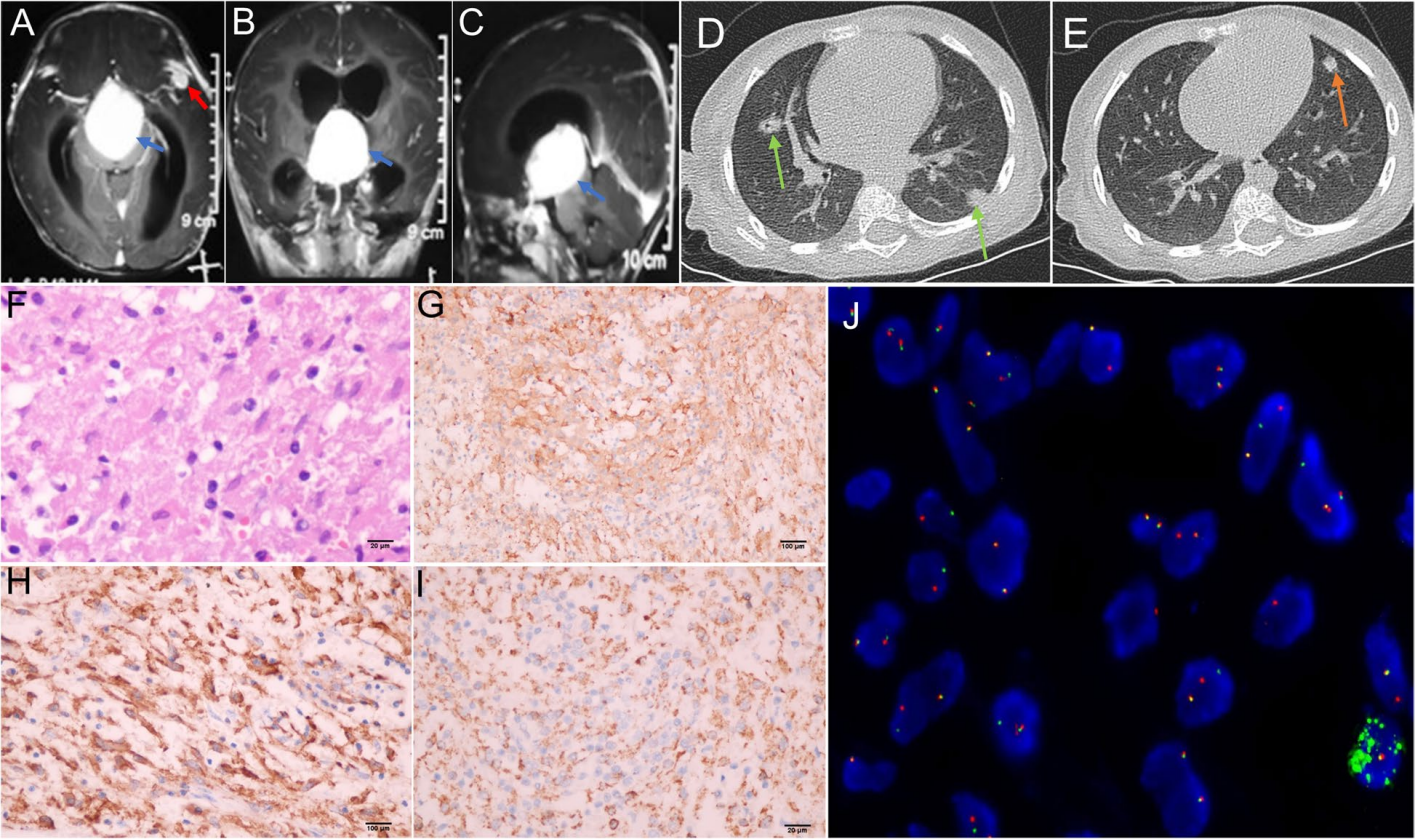
Imaging and pathological results of the case. The MRI of the head shows contrast-enhancing lesions involving the suprasellar region (**A**-**C**, blue arrow) and the left temporal lobe (**A**, red arrow). The lung CT reveals multiple nodes in the right lung (**D**, green arrow) and the left lung (**E**, brown arrow). Hematoxylin and eosin staining show an infiltrative proliferation of spindle cells (**F**). Immunostaining for ALK (**G**), CD68 (**H**), and CD163 (**I**) is positive. ALK rearrangement (green and red signals) is detected in the FISH test (**J**). The microscopy images was captured by multispectral automatic tissue quantitative scanning and analysis system and ZEISS Fully Automatic Upright Fluorescence Microscope.The measured resolution for images was 360 ppi

The laboratory examination results, including blood routine and blood biochemistry, were normal. The biological biomarker test revealed that the values of AFP and β-HCG were normal. The contrast-enhanced MRI of the head revealed inhomogeneous lesions with obvious enhancement in the suprasellar region and left middle cranial fossa, ventricular dilation, and peripheral brain edema (Fig. 2A-C). Multiple pulmonary nodules were visible in computed tomography (CT) before the start of systemic treatment (Fig. 2D-E).

Due to severe bleeding, we performed partial tumor resection. The pathological diagnosis of the lesions in the brain was confirmed through immunohistochemistry, high-throughput sequencing, and fluorescence in situ hybridization (FISH). The immunostaining result revealed that the positive terms were CD163, CD30, ALK-1, CD4, Cyclin D1, Ki67 (+ , 5%), and CD68/PGM1, while the negative terms were CXCL13, Langerin, EGFR, SSTR2, SALL4, and PLAP () (Fig. 2F-I). The result of the NGS genetic test revealed KIF5B-ALK gene rearrangement (fusion) (K24:A20) (Abundance: 21.59%). The capture-based high-throughput sequencing analysis did not identify any variation in gene copy numbers. Anaplastic lymphoma kinase gene translocation was confirmed by FISH (Fig. 2J).

**Fig. 3:**
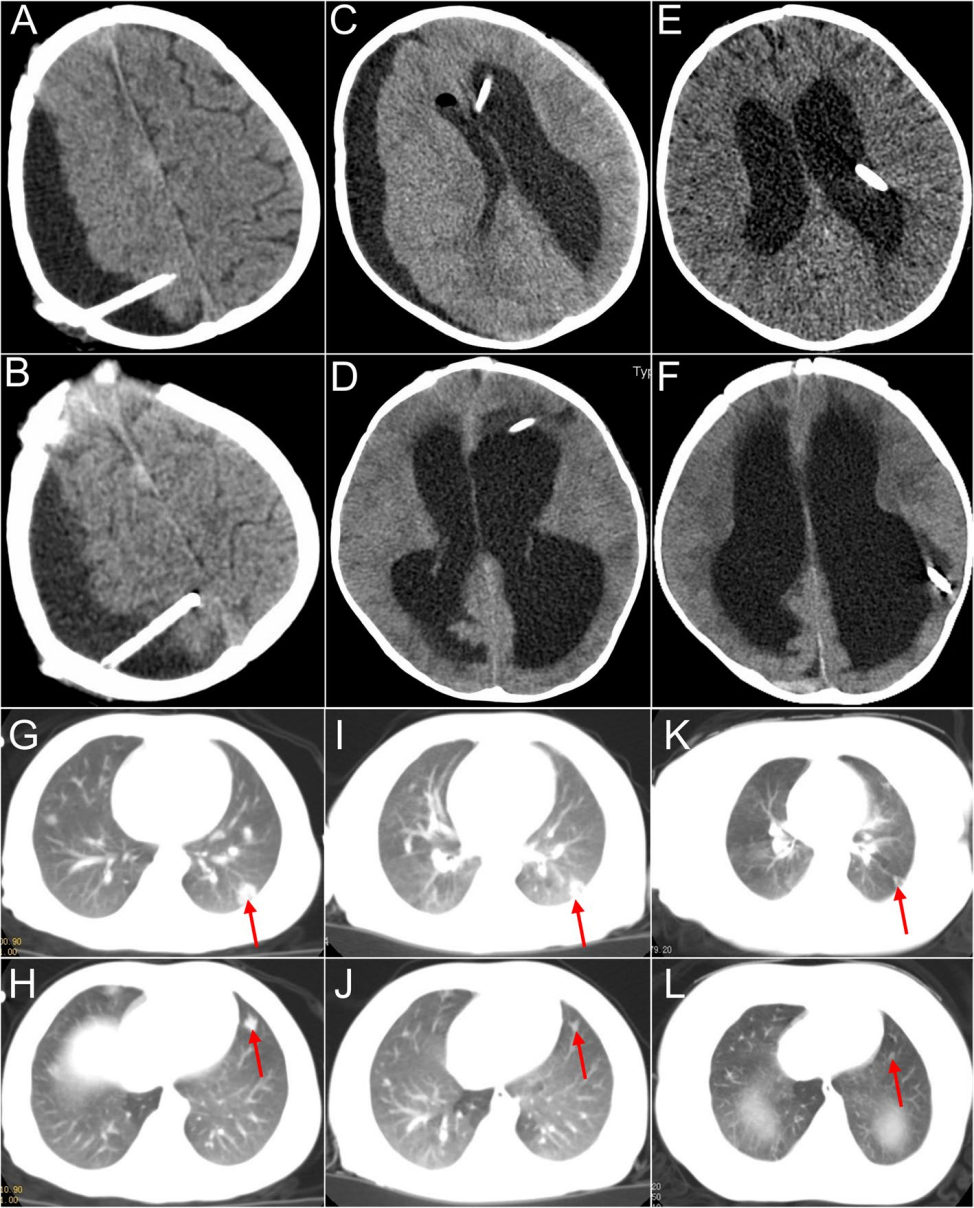
The management of CSF and the lung CT during chemotherapy. The SDE did not relieve on day 1 (**A**) and day 7 (**B**) after burr hole drainage. SE gradually relieved on day 1 (**C**) and day 7 (**D**) after implanting the Ommaya reservoir. SE gradually relieved on day 1 (**E**) and day 7 (**F**) after implanting a long-distance open ventricular drainage operation. The nodes (red arrow) on the lungs did not shrink one month (**G **and **H**) and two months (**I** and **J**) after the management of treatment with crizotinib and vincristine. After two months of cytarabine, crizotinib, and vincristine treatment, lesion remission was observed (**K** and **L**). The measured resolution for images was 360 ppi

Additionally, the head CT confirmed hydrocephalus and subdural effusion (SDE) on the right side of the patient. A burr hole drainage procedure was performed (Fig. 3A), but SDE did not relieve (Fig. 3B). After the drainage tube was removed, the consciousness of the patient deteriorated again. Moreover, both the glucose level and the number of white blood cells in cerebrospinal fluid (CSF) were higher than normal. For further treatment, the Ommaya reservoir was implanted in the left lateral ventricle (Fig. 3C), and CSF was aspirated daily through the Ommaya reservoir. The effusion on the right side decreased (Fig. 3D). Anti-infection medication ceftriaxone sodium was prescribed. Half a month later, the patient had a fever, and bacillus cereus was found in the CSF culture. In this case, meropenem was prescribed. Because the fever was not controlled and the bacillus cereus was positive in the CSF, we added vancomycin. Despite the relief of fever, CSF culture showed the presence of bacillus cereus. Bacillus cereus was suspected to be colonized in the Ommaya reservoir. Consequently, external ventricular drainage was performed first, followed by the removal of the Ommaya reservoir (Fig. 3E). The effusion on the right side also disappeared (Fig. 3F). Three consecutive CSF cultures were negative, and the body temperature was normal. We discontinued antibiotic therapy at this point.

**Fig. 4:**
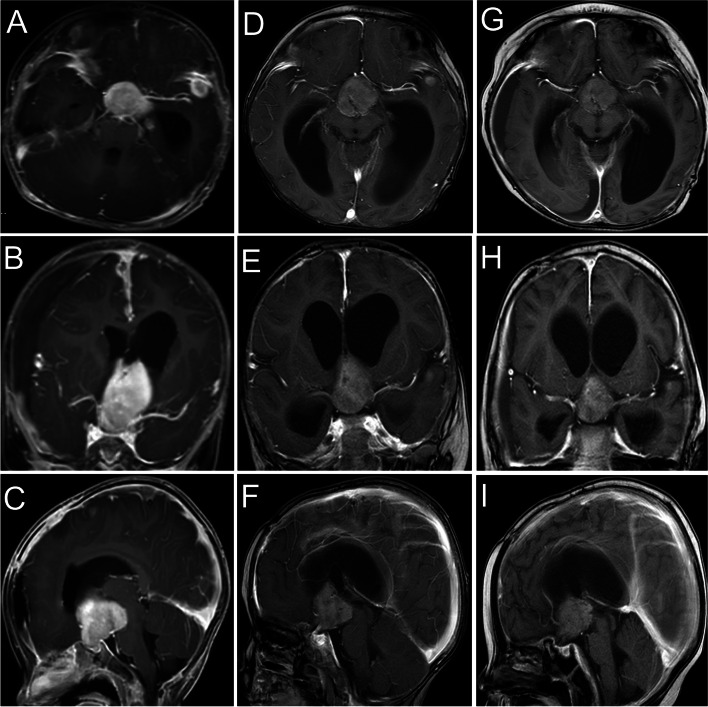
The MRI of the head during chemotherapy with the measurement of RECIST (The Response Evaluation Criteria In Solid Tumors). Compared with the lesions (**A**-**C**) after resection (2.4 × 3.1x2.6 cm), the lesion on MRI was stable two months after chemotherapy management with crizotinib and vincristine (**D**-**F**) (2.6 × 3.3x2.8 cm). After two months of treatment with cytarabine, crizotinib, and vincristine, tthe effect of the treatment was partial response (**G**-**I**) (2.4 × 2.3x2.1 cm). The measured resolution for images was 360 ppi

Approximately two months after the resection of lesions, the patient was given prednisolone acetate, oral crizotinib (80 mg per day, twice a day), and intravenous vincristine (0.55 mg per week). However, the lung CT showed that the pulmonary nodules did not shrink significantly after two months of chemotherapy (Fig. 3G-I). Additionally, there was no significant change in the size of the lesions in the brain (Fig. 4A-F).

**Fig. 5:**
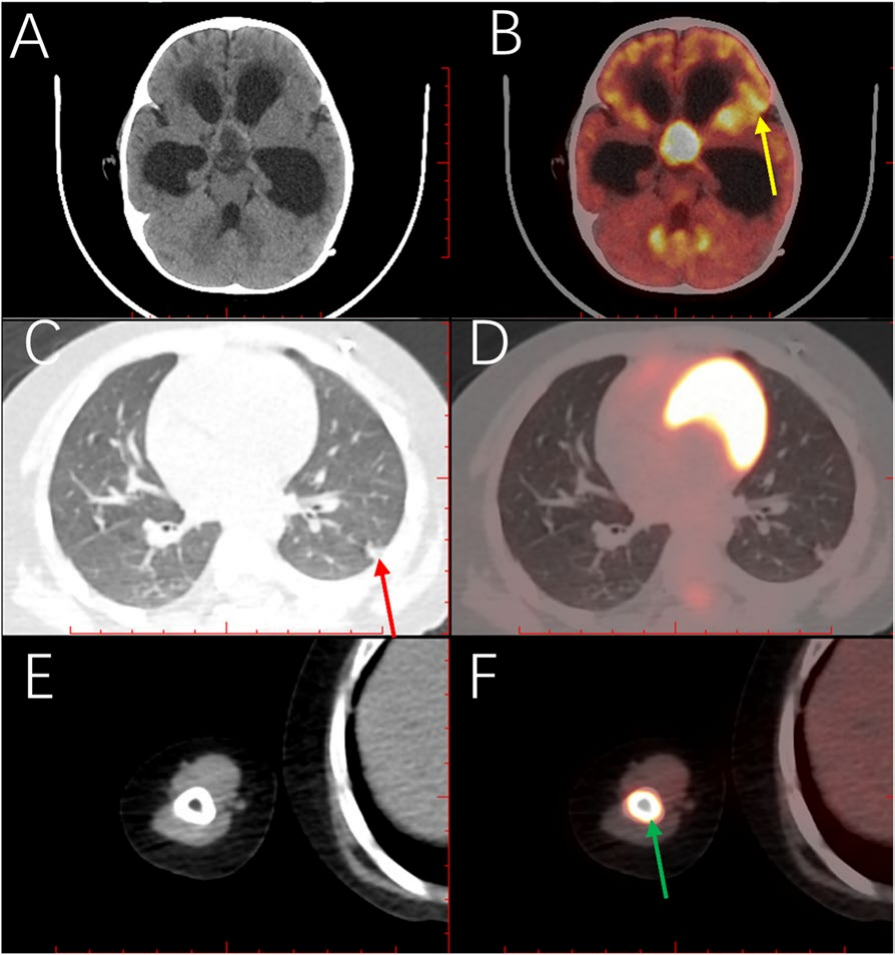
The whole-body positron emission tomography-computed tomography (PET-CT). PET-CT scan shows disseminated lesions involving multiple organs, including the brain (**B**, yellow arrow), the lung (**C**, red arrow), and the right humerus (**F**, green arrow). The measured resolution for images was 360 ppi

In addition, the PET-CT scan showed that the patient had a new lesion in the right humerus, in addition to for existing lesions in the suprasellar, lungs, and left middle cranial fossa (Fig. 5A-F). We suspected that the lesion might be a possibility of progression of disease. Multiple organs were affected by the ALK-positive histiocytosis. Then, the patient was given cytarabine (40 mg per day, five times in a row, two weeks apart) except for crizotinib and vincristine. The dosage of the crizotinib was increased from 80 mg per day to 100 mg per day, and prednisolone acetate was changed to dexamethasone. Imaging confirmed that the therapeutic regimen was effective (Figs. 3J-L and 4G-I) after application of the cytarabine for 40 days.Unfortunately, the patient passed away due to multiple ALK‑positive histiocytosis, hydrocephalus, subdural effusion, serious intracranial infection, deep vein thrombosis of the lower extremity, cachexia, and pneumonia after the application of crizotinib, dexamethasone, vincristine, and cytarabine for approximately two months.

## Literature review

According to the literature review, only KIF5B-ALK fusions found in the CNS,the effectiveness of gross total resection alone, localized or disseminated lesions, more common in Asians, and ALK inhibitors are the characteristics of lesions which involved the CNS (Table 1). Only eight cases of CNS involvement have been reported in the literature: three localized cases and five disseminated cases. These characteristics are not consistent with those of infants with systemic but self-limited disease and older children and adults with localized disease [6].

**Table 1:** Clinical characteristics of ALK-positive histiocytosis in the literature

No	Age	Sex	Position	Ethnicity	Localized or Disseminated	Surgery	Base mutation	Chemotherapy	Follow-up
1 [2]	15 years,	M	Cavernous sinus	Caucasian	Localized	No	KIF5B-ALK	ALK-inhibitor	No recurrence after 6 months
2 [3]	7 years,	F	Cerebellum	NA	Localized	Total resection	KIF5B-ALK	No	No recurrence after 12 months
3 [3]	10 years,	F	Cerebrum	NA	Localized	Total resection	KIF5B-ALK	No	No recurrence after 6 months
4 [14]	11 years	F	Right frontal lobe	NA	Disseminated	Total resection	KIF5B-ALK	No	No recurrence after 4 months
5 [14]	10 months	M	CNS, pulmonary, hepatic and peritoneal nodule	NA	Disseminated	Partial resection	KIF5B-ALK	ALK-inhibitor	Stable at 7 months
6 [12]	51 years	F	Lung, CNS	Asian	Disseminated	Tumor resection	KIF5B-ALK	ALK-inhibitor	Stable at 7 months
7 [2]	2 years, 9 months	M	Intestine, bone marrow, CNS	Middle Eastern	Disseminated	No	NA	etoposide, cyclosporine, immunoglobulins, cytarabin, methotrexate	Died after 2 months
8 [7]	49 years	M	CNS, bone, soft tissue, visceral organs, pleura	Caucasian	Disseminated	No	KIF5B-ALK	Gamma knife, lenalidomide, pembrolizumab, ALK inhibitor	Stable at 7 months
9^**a**^	1 year, 4 months	M	Suprasellar	Chinese	Disseminated	Partial resection	KIF5B-ALK	ALK-inhibitor + cytarabine + vincristine	Six months

*M* Mmale, *F* Female, *CNS* Central nervous system, *NA* Not available

^a^our case

## Discussion and conclusions

To our knowledge, lesions involving mesentery, breast, appendix, extremity, peripheral blood, kidney, bone marrow, lung, brain, and lymph nodes have been reported since the advent of ALK-positive histiocytosis in 2008. Some are local lesions, and some are part of systemic lesions [2–13]. However, no lesions in the suprasellar region have been reported. In this study, we first reported the case of a one-year-and-four-month-old boy with ALK-positive histiocytosis in the suprasellar region. After combining the high-quality results of large international collaboration on ALK-positive histiocytosis with our case [14], we believe that this case may provide a reference for patients involving CNS.

Confirmative diagnosis is primarily based on neuropathological screening, which includes the determination of tissue features, immunohistochemical assay, and genetic mutation testing for ALK translocation. Moreover, an accurate pathological diagnosis of ALK-positive histiocytosis can guide treatment. Due to its rarity and the overlapping morphological features with Erdheim-Chester disease (ECD), juvenile xanthogranuloma, Rosai-Dorfman disease (RDD), and Langerhans cell histiocytosis (LCH), the pathological differential diagnosis of this disease is extremely challenging [15]. The features of these entities are shown in Table 2. The presentation, morphology, and immune profile of each disease are helpful in the differential diagnosis. Microscopically, ALK-positive histiocytosis is characterized by large epithelioid cells, Touton-like giant cells, absence of substantial atypia [6], and focal emperipolesis. The immunohistochemical assay shows ALK, CD68, and CD163, but not CD1a, BRAFV 600E, and GFAP. In our case, CD68, CD163, and ALK were positive, so we suspected that the patient had ALK-positive histiocytosis. ALK-positive histiocytosis accompanied by diffuse cytoplasmic positivity of S-100 protein may be mistaken for RDD [9]. However, mutations in the RAS pathway are only found in RDD [13]. Moreover, plasma cells are rare in ALK-positive histiocytosis. In the absence of a BRAF mutation, it is difficult to distinguish ECD from ALK-positive histiocytosis. KIF5B-ALK fusion has also been reported in three adult cases of ECD with disseminated disease [16, 17]. A lack of skeletal involvement and xanthomatous foamy histiocytes may rule out ECD in this case. When KIF5B-ALK fusion is present in juvenile xanthogranuloma (JXG) [18], foamy histiocytes with S-100 protein can be different from ALK-positive histiocytes. A CD1a immunostain can rule out LCH. Thus, high-throughput sequencing and FISH were performed to confirm that the disease was ALK-positive histiocytosis.

**Table 2:** The differential diagnosis of ALK-positive histiocytosis with different entity

Entity	Mutation style	Positive marker in IHC	Morphologic features	Negative marker in IHC
ECD	KIF5B-ALK fusion, the uncommon BRAF V600E mutation	CD68, CD163	Foamy histiocytes with small nuclei and Touton giant cells	CD1a, positive S100 in some histiocytes, langerin,
JXG	KIF5B-ALK fusion	S100, CD11c, CD4	Touton giant cells, oval nuclei in foamy histiocytes	CD1a,
RDD	Mutations in the RAS pathway	S100, CD68	Round nuclei, vesicular chromatin, distinct Nucleoli	CD 1a
LCH	BRAF V600E mutation in t 50– 65% of patients	S-100, CD1a, CD207	Nuclear convolutions, vesicular nuclei, large cytoplasm	CD68, CD163
ALK-positive histiocytosis	KIF5B-ALK fusion	ALK, CD68, CD163, XIIIa,	Large epithelioid cells, Touton-like giant cells, absence of substantial atypia	CD1a, BRAFV600E, GFAP

*ECD* Erdheim-Chester disease, *JXG* Juvenile xanthogranuloma, *RDD* Rosai-Dorfman disease, *LCH* Langerhans cell histiocytosis

Mutations of ALK-positive histiocytosis genes include KIF5B-ALK, TPM3-ALK, COL1A2-ALK, TRIM33-ALK, and EML4-ALK [2–13]. However, the only documented fusion of ALK-positive histiocytosis in CNS is KIF5B-ALK [2, 3, 7, 12, 15]. There appears to be no relationship between localization or dissemination of ALK-positive histiocytosis in the CNS and KIF5B-ALK fusion. Therefore, identifying the ALK mutation is vital. KIF5B and ALK encode the ubiquitous isoform of the heavy chain of kinesin-1 and a receptor tyrosine kinase, respectively [19]. In ALK-positive histiocytosis, the KIF5B-ALK fusion may lead to targetable kinase alterations as oncogenic drivers [16].

The treatment strategies for ALK-positive histiocytosis involving the CNS should be specific. Lesion resection can relieve the symptom. The biopsy has low risk and yields substantial information for the confirmative diagnosis. To treat a local primary CNS lesion, only gross total resection may be needed without an ALK inhibitor [2, 3, 15]. An ALK inhibitor may be necessary to control the disease involving the CNS of the disseminated lesion [2, 7, 12, 15]. Even though lesion decompression was used to relieve symptoms and ALK inhibitors were prescribed for adjuvant therapy, the lesion size did not change significantly. In addition, the lesions on the right humerus might be possibility of progression of disease. The poor effect might be caused by the poor penetration of the brain-blood barrier, the big size of the lesions in the brain, multiple lesions, and the weak constitution and intracranial infection due to the several surgeries. At present, many ALK inhibitors have an excellent ability to penetrate the blood–brain barrier. Crizotinib has a limited CNS passage to penetrate the blood–brain barrier [20]. However, the application of these medications, such as alectinib, ceritinib and lorlatinib, can increase the brain-to-blood exposure ratio [21]. Another ALK inhibitor, alectinib, is effective in treating disseminated ALK-positive histiocytosis in CNS [12].

In addition, managing surgery complications, such as hydrocephalus, SDE, and intracranial infection after surgery, can be challenging. A subdural effusion with hydrocephalus (SDEH) has been reported in cases of foramen magnum decompression and clipping of intracranial aneurysms after surgery [22, 23]. Several successful cases with the ventricle drainage tube implanted have been reported [23–25], as in the present case. Finding the site of bacterial colonization is crucial. In our case, symptoms of an intracranial infection were relieved after the drainage tube and Ommaya reservoir were removed.

In general, the present study reported the case of a one-year-and-four-month-old boy with ALK-positive histiocytosis involving the suprasellar region. The adjuvant chemotherapy drugs are needed when ALK-inhibitor treatment is ineffective in treating the lesion. The SDEH may be relieved with the implantation of ventricle drainage. The disease in the CNS is characterized by only KIF5B-ALK fusion, the effectiveness of gross total resection alone, localized or disseminated lesion, more common in Asians, and efficacy of ALK-inhibitor treatment.

## Data Availability

The original contributions presented in the study are included in the article. Further inquiries can be directed to the corresponding authors.

## Abbreviations

ALK: Anaplastic lymphoma kinase
CNS: Central nervous system
NGS: Next-generation sequencing
MRI: Magnetic resonance imaging
CT: Computed tomography
FISH: Fluorescence in situ hybridization
SDE: Subdural effusion
CSF: Cerebrospinal fluid
ECD: Erdheim-Chester disease
LCH: Langerhans cell histiocytosis
SDEH: Subdural effusion with hydrocephalus
